# Simulating PACE Global Ocean Radiances

**DOI:** 10.3389/fmars.2017.00060

**Published:** 2017-03-06

**Authors:** Watson W. Gregg, Cécile S. Rousseaux

**Affiliations:** NASA Global Modeling and Assimilation Office, Greenbelt, MD, USA

**Keywords:** PACE, ocean color, water-leaving radiances, biogeochemical model, radiative transfer model

## Abstract

The NASA PACE mission is a hyper-spectral radiometer planned for launch in the next decade. It is intended to provide new information on ocean biogeochemical constituents by parsing the details of high resolution spectral absorption and scattering. It is the first of its kind for global applications and as such, poses challenges for design and operation. To support pre-launch mission development and assess on-orbit capabilities, the NASA Global Modeling and Assimilation Office has developed a dynamic simulation of global water-leaving radiances, using an ocean model containing multiple ocean phytoplankton groups, particulate detritus, particulate inorganic carbon (PIC), and chromophoric dissolved organic carbon (CDOC) along with optical absorption and scattering processes at 1 nm spectral resolution. The purpose here is to assess the skill of the dynamic model and derived global radiances. Global bias, uncertainty, and correlation are derived using available modern satellite radiances at moderate spectral resolution. Total chlorophyll, PIC, and the absorption coefficient of CDOC (a_CDOC_), are simultaneously assimilated to improve the fidelity of the optical constituent fields. A 5-year simulation showed statistically significant (*P* <0.05) comparisons of chlorophyll (*r* = 0.869), PIC (*r* = 0.868), and a_CDOC_ (*r* = 0.890) with satellite data. Additionally, diatoms (*r* = 0.890), cyanobacteria (*r* = 0.732), and coccolithophores (*r* = 0.716) were significantly correlated with in situ data. Global assimilated distributions of optical constituents were coupled with a radiative transfer model (Ocean-Atmosphere Spectral Irradiance Model, OASIM) to estimate normalized water-leaving radiances at 1 nm for the spectral range 250–800 nm. These unassimilated radiances were within −0.074 mW cm^−2^ μm^1^ sr^−1^ of MODIS-Aqua radiances at 412, 443, 488, 531, 547, and 667 nm. This difference represented a bias of −10.4% (model low). A mean correlation of 0.706 (*P* < 0.05) was found with global distributions of MODIS radiances. These results suggest skill in the global assimilated model and resulting radiances. The reported error characterization suggests that the global dynamical simulation can support some aspects of mission design and analysis. For example, the high spectral resolution of the simulation supports investigations of band selection. The global nature of the radiance representations supports investigations of satellite observing scenarios. Global radiances at bands not available in current and past missions support investigations of mission capability.

## INTRODUCTION

The now 19-year time series of routine global ocean color observations from space has led to advancements in the science of ocean biology beyond expectations. From chlorophyll interannual variability to inherent optical properties to physical-biological coupling, the time series has been an invaluable resource for scientists in a broad range of ocean and atmosphere-related fields. As is often the case in science, the proliferation of information from these moderate resolution missions has raised as many questions as it has answered. Coupled with improvements in detector technology, the time is now right for advancement of ocean biogeochemical science from space using higher spectral resolution missions.

Higher spectral resolution can potentially improve detection of optical constituents in the oceans that have important effects on biology, biogeochemistry, and light transmission. One major objective is the determination of phytoplankton groups from space. Research to detect phytoplankton groups from space has been going on for some time using the fleet of moderate spectral resolution sensors (e.g., Kamykowski et al., 2002; Alvain et al., 2005; Aiken et al., 2007; Bracher et al., 2009; Brewin et al., 2010, 2011; Kostadinov et al., 2010; Masotti et al., 2010; Hirata et al., 2011). Methods to identify size classes have also been pursued (e.g., Loisel et al., 2006; Brewin et al., 2011) but these only loosely relate to phytoplankton functionality/taxonomy. Several phytoplankton discrimination methods resolve dominant groups only (Sathyendranath et al., 2004; Alvain et al., 2005, 2008; Hirata et al., 2008; Raitsos et al., 2008). Hirata et al. (2011) provides taxonomic classifications, with relative and even absolute abundances quantified. Using satellite ocean chlorophyll concentrations rather than radiances, this empirical methodology essentially assumes that abundance reflects taxonomy, which is valid in many instances but not always (Rousseaux et al., 2013).

Moderate resolution ocean color sensors containing only a few discrete spectral bands, such as the global missions flown to date, do not contain sufficient spectral information to enable unequivocal phytoplankton functional/taxonomic discrimination. Many phytoplankton species/groups have subtle, but distinct spectral signatures. Use of hyper-spectral remote retrievals with many bands spanning the visible and ultraviolet spectrum holds potential for resolving these spectral distinctions (e.g., Bracher et al., 2009; Sadeghi et al., 2012; Palacios et al., 2015; Neukermans et al., 2016).

To close this knowledge gap, NASA has proposed the PACE mission, a global hyper-spectral sensor to test the ability to retrieve phytoplankton population distributions, as well as other important ocean constituents with optical signatures. The mission, proposed for launch in the early 2020’s, can potentially demonstrate the feasibility and capability of hyper-spectral observations from space and enable scientists to observe and quantify these important ocean biological features. PACE is intended to follow future planned hyperspectral missions PRISM (Meini et al., 2015) and EnMAP (Foerster et al., 2015) with extended spectral range into the ultraviolet, faster observational repeat times, and emphasis on global ocean observational capability.

Since there is no global observational precedent, many mission development activities, design tradeoff assessments, operational strategies, and other issues, are speculative. Here we develop a dynamic global model at extreme hyper-spectral resolution (1 nm) to provide a platform to approximate realistic ocean conditions and help with resolving at least some of these issues and understand if such a simulation can assist in resolving many of the issues that inevitably arise in the design and testing of a new mission. The objective of this effort is to quantitatively assess the skill of a global model using a forward radiance representation to simulate global ocean water-leaving radiances. The skill is evaluated spectrally with explicit error characterization.

## METHODS

### Global Ocean Physical-Biogeochemical Model Configuration

The underlying biogeochemical constituents are simulated by the NOBM which is coupled to a global ocean circulation model, Poseidon (Schopf and Loughe, 1995). It spans the domain from −84° to 72° latitude in increments of 1.25° longitude by 2/3° latitude, including only open ocean areas, where bottom depth >200m. NOBM incorporates global coupled physical-biological processes, including four phytoplankton groups (diatoms, chlorophytes, cyanobacteria, and coccolithophores), which span much of the functionality of the global oceans, four nutrients (nitrate, ammonium, silicate, and dissolved iron), three detrital components (particulate organic carbon, silicate, and iron), and two carbon components (dissolved organic and inorganic carbon). It is a three-dimensional representation of coupled circulation/biogeochemical processes in the global oceans (Gregg et al., 2003; Gregg and Casey, 2007).

Optically-active constituents have been added to NOBM to improve realism and complexity of the ocean simulation and better represent the ocean optical variability that will be observed by PACE. We have added particulate inorganic carbon (PIC) and chromophoric dissolved organic carbon (CDOC) as prognostic state variables. PIC is produced by coccolithophores as detached coccoliths and is lost via sinking and dissolution. PIC is produced as a fraction (25%) of the coccolithophore growth rate (Gregg and Casey, 2007) minus respiration. The PIC sinking rate is represented here as an exponential function of concentration, assuming that large concentrations of PIC are associated with larger coccolith size.

(1) $$w_{s}(\text{PIC})=a_{o}\exp(a_{1}*\text{PIC})$$

where w_s_ is the PIC sinking rate (m d^−1^), PIC is in units of μgC l^−1^, a_0_ = 0.1 m d^−1^ and a_1_ = 2.0 l μgC^−1^ (Gregg and Rousseaux, 2016). Dissolution follows Buitenhuis et al. (2001), except that no dissolution is allowed for depths shallower than the calcium carbonate compensation depth, which we define as 3500m.

Chromophoric dissolved organic carbon (CDOC) represents the biogeochemical constituent necessary for the simulation of absorption by a_CDOC_(λ), the absorption coefficient, which is an optical quantity. CDOC is formed and destroyed the same as DOC, using Aumont et al. (2002) with an assumed DOC:CDOC production/loss ratio of 0.5. It is additionally destroyed by the absorption of spectral irradiance. We follow the methodology of Gregg and Rousseaux (2016) for photo-destruction (photolysis) of CDOC per unit irradiance quanta, with a different quantum yield φ*CDOC* of 3.0E-6 (μM μmol photons absorbed m^−3^) for results in reasonable agreement with MODIS-Aqua data (Maritorena et al., 2010).

### Ocean-Atmosphere Spectral Irradiance Model

**FIGURE 1:**
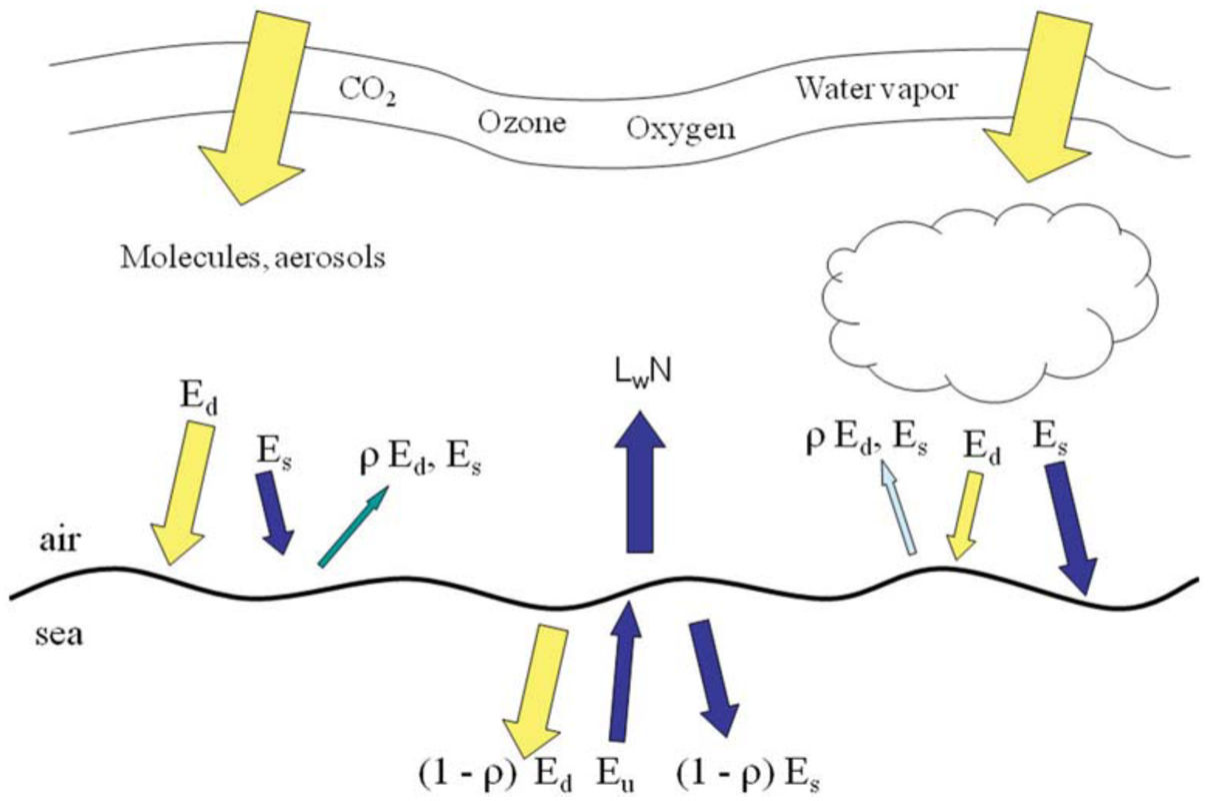
irradiance pathways in OASIM E_d_ is direct downwelling irradiance, E_s_ is diffuse downwelling. ρ surface reflectance, E_u_ is diffuse upwelling irradiance, and L_w_N is normalized water-leaving radiance. All irradiances and radiances are spectrally resolved at 25 nm for E_d_, E_s_, and E_u_. and 1 nm for L_w_N.

NOBM is coupled to OASIM (Gregg and Carder, 1990; Gregg, 2002; Gregg and Casey, 2009) to simulate the propagation of downward spectral irradiance in the oceans and the upwelling irradiance/radiance. The irradiance pathways for OASIM are shown in Figure 1. The atmosphere and ocean portions of the downwelling and upwelling irradiance are implemented at 25-nm spectral resolution. Higher spectral resolution is impractical for global models that integrate at 30 min time steps in our case. Upwelling radiance is produced at 1 nm resolution, however. Biases and uncertainties in the atmospheric component of OASIM have been characterized for clear sky high spectral resolution (1 nm; Gregg and Carder, 1990) and under mixed cloudy and clear skies for integrated spectral resolution (Gregg and Casey, 2009). We elaborate here on the ocean optical calculations.

### Optical Properties of Ocean Constituents

**FIGURE 2:**
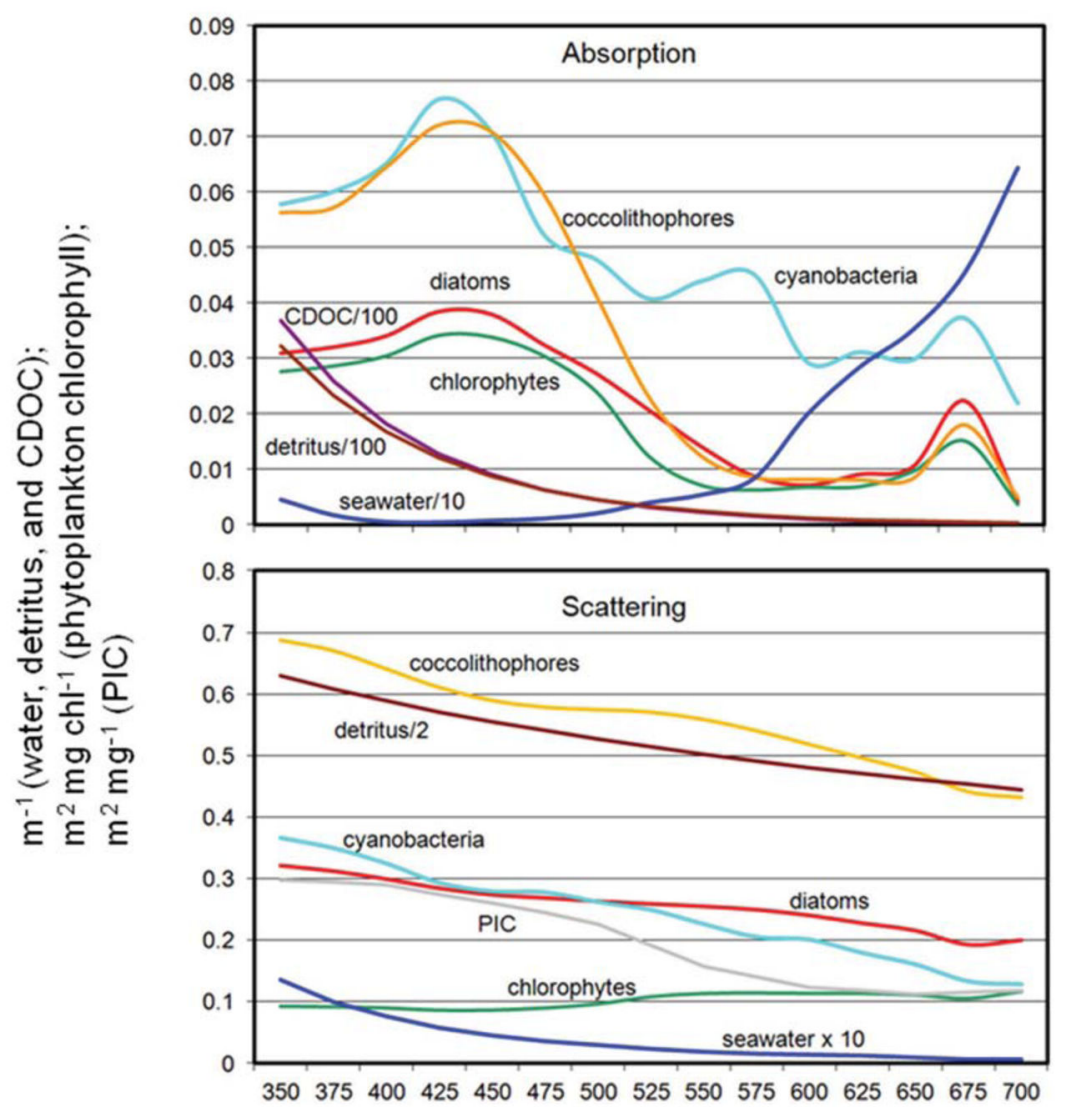
Spectral absorption and scattering coefficients of water, phytoplankton, detritus, PIC, and CDOC in OASIM.

The coupled NOBM-OASIM model includes optically active constituents, including seawater, phytoplankton, detritus, PIC, and CDOC each with unique spectral characteristics (Figure 2). All are prognostic state variables, with independent sources and sinks. The optical properties of each constituent are taken from various efforts in the peer reviewed literature.

#### Water

The spectral absorption and scattering properties of seawater was reported by Smith and Baker (1981) for the 200–800 nm spectral domain. Pope and Fry (1997) revised this for the range 380–720 nm, but this was for pure water. Morel et al. (2007) derived new data for absorption and scattering for the spectral range 300–500 nm using information in the clearest ocean waters of the South Pacific (although absorption values >420 nm were taken from Pope and Fry, 1997). Finally, Lee et al. (2015) reported absorption coefficients in the range 350–550 nm derived using remote sensing reflectance algorithms for the same clear ocean water data used by Morel et al. (2007). Mason et al. (2016) used laboratory observations to obtain new absorption coefficients for the spectral range 250–550 nm. Like Pope and Fry (1997), their results were specific to pure water.

Water absorption data used here are from Smith and Baker (1981) for 200–300 nm and 730–800 nm, Morel et al., 2007) for 300–350 nm, Lee et al. (2015) for 350–550 nm, Pope and Fry (1997) for 550–720 nm, Circio and Petty (1951) for 800 nm–2.5 μm, and Maul (1985) for 2.5–4 μm. Water scattering is from the method of Zhang et al. (2009), which accounts for temperature and salinity dependence. The backscattering-to-total scattering ratio b_bw_ for water is 0.5.

#### Phytoplankton

Phytoplankton optical properties are obtained from various sources. Chlorophyll-specific absorption coefficients a^*^_p_(λ) are derived by taking reported spectra and normalizing to the absorption at 440 nm [a^*^_p_(440)]. Normalized specific absorption spectra [a^*^_p_(λ)]_N_ are computed for each of the four phytoplankton groups: diatom and chlorophyte [a^*^_p_(λ)]_N_ are taken from Sathyendranath et al. (1987), cyanobacteria from Bricaud et al. (1988), and coccolithophores from Morel and Bricaud (1981). Then the specific spectral a^*^_p_(λ) values are derived using mean values at 440 nm. Diatom a^*^_p_(440) represents the mean of 5 observations containing 4 different spp., chlorophytes 6 observations from 4 spp., cyanobacteria 5 observations from 3 spp., and coccolithophores 3 observations of 1 spp.

Phytoplankton specific scattering coefficients b^*^_p_(λ) are obtained from measurements at 590 nm and extended to the entire spectrum from specific attenuation coefficients (Bricaud et al., 1988). Diatom and chlorophyte specific scattering coefficients at 590 nm, b^*^_p_(590) and b*_p_(590), are the mean of 5 observations and 6 observations, respectively, from Morel (1987), Bricaud and Morel (1986), and Bricaud et al. (1988). Cyanobacteria b^*^_p_(590) is the mean of 8 observations from Morel (1987), Bricaud and Morel (1986), Bricaud et al. (1988), and Ahn et al. (1992). Coccolithophore b^*^_p_(590) is derived from the mean of 3 observations from Bricaud and Morel (1986), Bricaud et al. (1988), and Ahn et al. (1992).

We assume no spectral dependence in the backscattering-to-total scattering ratio b̃_bp_. Ahn et al. (1992) suggested a spectral dependence for cyanobacteria but generally none for the other groups. Reported values for b̃_bp_ are 0.002 for diatoms (Morel, 1988), 0.00071 for chlorophytes, 0.0032 for cyanobacteria (Ahn et al., 1992), and 0.00071 for coccolithophores (Morel, 1988). Some of these values have come under question based on non-sphericity of many natural phytoplankton populations (Vaillancourt et al., 2004; Whitmire et al., 2010). Based on these results, we increased b̃_bp_ for chlorophytes and coccolithophores by a factor of 10, but kept them as reported for diatoms and cyanobacteria.

#### Detritus

Detritus both absorbs and scatters light (Figure 2). Absorption is typically considered an exponential function of wavelength (Roesler et al., 1989; Gallegos et al., 2011).

(2) $$a_{d}(\lambda )={\text{Da}}_{d}^{*}\exp[-S_{d}(\lambda -440)]$$

where a_d_(λ) is the absorption coefficient of detritus (m^−1^), D is the concentration of detritus μg C m^−3^, S_d_ = 0.013 nm^−1^ (Gallegos et al., 2011) and $a_{d}^{*}$ is the mass-specific absorption coefficient of detritus, which is set to 8.0E-5 m^2^ mg^−1^ for small detritus as typically found in oceanic waters (Gallegos et al., 2011). Only organic carbon detritus in the model is used for detrital optics.

Detritus scattering is also taken from Gallegos et al. (2011).

(3) $$b_{d}(\lambda )=D\;b_{d}^{*}{\left(550/\lambda \right)}^{0.5}$$

where b_d_ is the total scattering coefficient, and b*_d_ is the mass-specific scattering coefficient, which is set as 0.00115 m^2^ mg^−1^, and the backscattering-to-total scattering ratio b̃_bd_ is 0.005.

#### PIC

PIC optical properties have been evaluated by Gordon et al. (2009). We adopt this formulation for our simulation. PIC scatters irradiance but does not absorb

(4) $$b_{\text{PIC}}(\lambda )=\text{PIC}\;b_{\text{PIC}}^{*}(\lambda )$$

where PIC is the concentration of PIC (mgC m^−3^) and b^*^_PIC_(λ) is PIC-specific spectral scattering coefficient from Gordon et al. (2009) in units of m^2^ mgC^−1^. The backscattering-to-total scattering ratio b̃_bpic_ is from Balch et al. (1996), using their lower bound of 0.01.

#### CDOC

As a dissolved component, CDOC only absorbs and does not scatter. Its spectral absorption is similar to detritus but with a different slope

(5) $$a_{\text{CDOC}}(\lambda )=a_{\text{cdoc}}^{*}\exp[-S_{\text{cdoc}}(\lambda -443)]$$

where $a_{\text{cdoc}}^{*}$ is the mass-specific absorption coefficient of CDOC (m^2^ mg^−1^), S_cdoc_ = 0.014 nm^−1^ (Bricaud et al., 1981, 2010). S is in the low end range of observations in surface waters of the Equatorial Atlantic (Andrew et al., 2013) but only slightly lower than those observed in the Mediterranean Sea (Organelli et al., 2014). There are few reports of the mass-specific absorption coefficient of CDOC $a_{\text{cdoc}}^{*}$. We have found three observations in the literature (Carder et al., 1989; Yacobi et al., 2003; and Tzortziou et al., 2007). The more recent two are in agreement at 2.98 × 10^−4^ m^2^ mg^−1^ in 4 rivers in Georgia, USA (Yacobi et al., 2003) and 2.78 × 10^−4^ m^2^ mg^−1^ as the mean of 4 stations in the Rhode River, Maryland, USA (Tzortziou et al., 2007). Carder et al. (1989) reported a mean over about nearly an order of magnitude lower in the Gulf of Mexico (4.74 × 10^−5^ m^2^ mg^−1^). We choose Yacobi et al. (2003) for our simulation.

### Upwelling Spectral Radiance

OASIM uses 25-nmspectral resolution in the 350–7700 nm range in the coupled model for downwelling and upwelling irradiance needed for phytoplankton growth and CDOC destruction. For enhanced realism of the PACE simulation of upwelling radiance we increase the spectral resolution to 1 nm. Since all of the optical properties data are available at 5 nm resolution or less, it is reasonable to simply interpolate the 5 nm data. The computation of upwelling spectral radiance L_w_N(λ) is derived from the coupled expressions of downwelling and upwelling irradiance by Aas (1987) as modified by Ackleson et al. (1994).

(6) $$\frac{{\text{dE}}_{d}(\lambda )}{\text{dz}}=-C_{d}(\lambda )E_{d}(\lambda )$$

(7) $$\frac{{\text{dE}}_{s}(\lambda )}{\text{dz}}=-C_{s}(\lambda )E_{s}(\lambda )+B_{u}(\lambda )E_{u}(\lambda )+F_{d}(\lambda )E_{d}(\lambda )$$

(8) $$\frac{{\text{dE}}_{u}(\lambda )}{\text{dz}}=-C_{u}(\lambda )E_{u}(\lambda )-B_{s}(\lambda )E_{s}(\lambda )-B_{d}(\lambda )E_{d}(\lambda )$$

where E_d_(λ) is the spectral downwelling direct irradiance at the bottom of a model layer, E_s_(λ) is the downwelling diffuse irradiance, and E_u_(λ) is the upwelling diffuse irradiance. The attenuation terms C_x_ (where x is an indicator for the irradiance pathway d for direct downwelling, s for diffuse downwelling, and u for diffuse upwelling), backscattering terms B_x_, and forward scattering F_x_ differ for each of the irradiance pathways because of different shape factors (Aas, 1987; Ackleson et al., 1994) and mean cosines.

(9) $$C_{d}(\lambda )=[a(\lambda )+b(\lambda )]/{\underline{\mu }}_{d}$$

(10) $$C_{s}(\lambda )=[a(\lambda )+r_{s}b_{b}(\lambda )]/{\underline{\mu }}_{s}$$

(11) $$C_{u}(\lambda )=[a(\lambda )+r_{u}b_{b}(\lambda )]/{\underline{\mu }}_{u}$$

(12) $$B_{d}(\lambda )=b_{b}(\lambda )/{\underline{\mu }}_{d}$$

(13) $$B_{s}(\lambda )=r_{s}b_{b}(\lambda )/{\underline{\mu }}_{s}$$

(14) $$B_{u}(\lambda )=r_{u}b_{b}(\lambda )/{\underline{\mu }}_{u}$$

(15) $$F_{d}(\lambda )=(1-b_{b}^{\prime })b(\lambda )/{\underline{\mu }}_{d}$$

where a is the absorption coefficient, b is the total scattering coefficient, b_b_ is the backscattering coefficient, $b_{b}^{\prime }$ is the ratio of backscattering to total scattering, and μ is the mean cosine (constant for diffuse irradiance, but varies with solar zenith angle for direct irradiance). The shape factors are indicated by the r_x_ terms, and are specified as in Ackleson et al. (1994). Equation 5 can be solved a priori, which can then be used as a boundary condition, greatly simplifying the solution of the coupled Equations 6, 7.

Equation 8 can be simplified for normalized upwelling radiance since by its definition the surface downwelling irradiance does not include attenuation effects of the atmosphere and the solar zenith angle is assumed to be 0° with overhead sun (Gordon, 1997). Substituting the mean extraterrestrial irradiance (Thuillier et al., 2004) for downwelling irradiance, we can obtain upwelling normalized water-leaving radiance solving the Aas (1987) expressions and correcting for surface reflectance.

(16) $$L_{w}N(\lambda )=F_{o}(\lambda ,0^{-})(1-\rho )/(n^{2}Q)$$

where F_o_ is the mean extraterrestrial irradiance (mW cm^−2^ μm^−1^) just below the ocean surface (0^−^) derived using Aas (1987), ρ is the surface reflectance (0.021), n is the index of refraction (1.341) and Q is the radiance:irradiance distribution function (= π for normalized surface irradiance).

**FIGURE 3:**
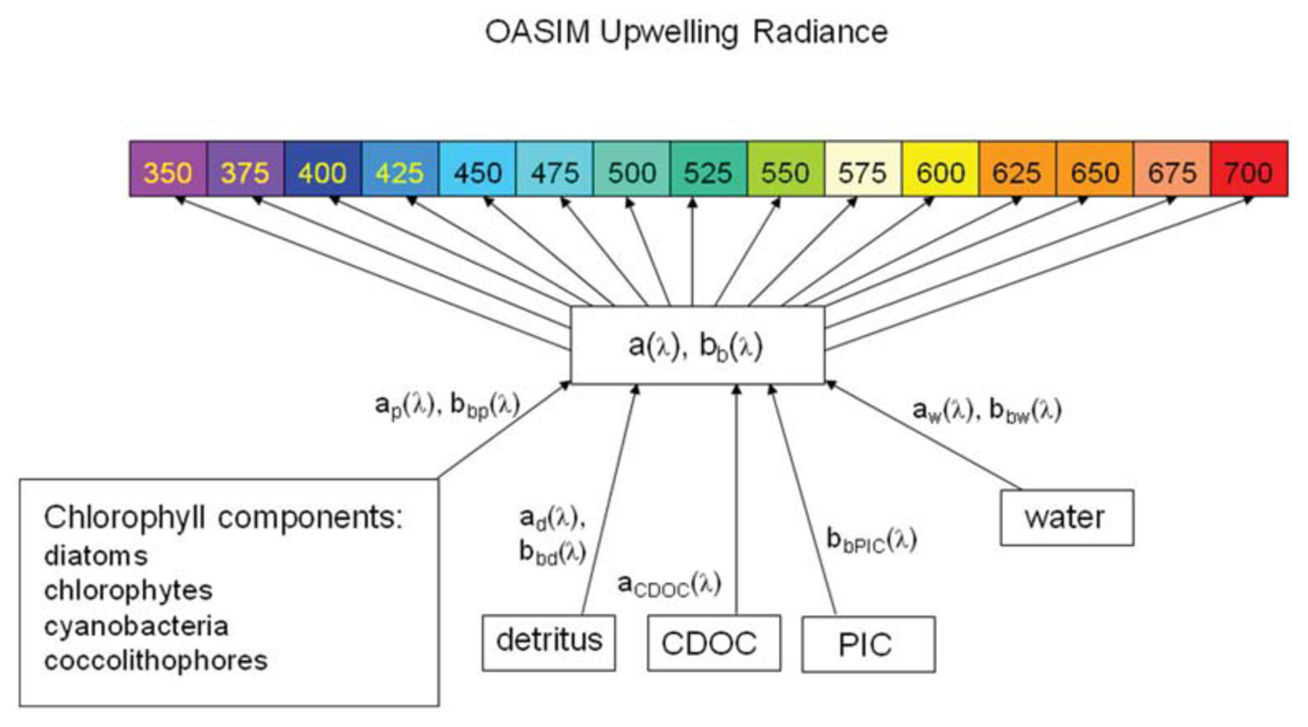
OASIM spectral upwelling radiance and dependencies in the ocean Shown are the visible bands. The spectral resolution for upwelling radiance is 1 nm. Inherent optical properties are derived from spectral characteristics of water, phytoplankton groups, detritus. PIC, and CDOC.

Using 1 nm spectral resolution L_w_N not only supports testing PACE sensor and mission concepts, it also simplifies comparison with MODIS-Aqua L_w_N by virtue of avoiding band mismatches. The pathways of optical constituents to optical properties to upwelling normalized water-leaving radiances as represented by the NOBM-OASIM global coupled physical-biogeochemical-optical model is depicted in Figure 3.

### Data Assimilation

Global total chlorophyll from MODIS is assimilated into NOBM using the method described in Gregg (2008). Additionally, global PIC from MODIS (Balch et al., 2005) is assimilated, using the same methodology except that the data are not log-transformed before assimilation. CDOC is assimilated, however, it requires a transformation before the process is executed. There is no available satellite data for CDOC, but a satellite product called a_CDM_ is available (Garver and Siegel, 1997; Maritorena and Siegel, 2005; Maritorena et al., 2010). We use the products from MODIS-Aqua in this effort. This product represents the absorption of both CDOM and detritus (hence the usage of CDM to minimize confusion about its nature). Siegel et al. (2002) estimated the detrital contribution as 12%. We assume this is globally constant and apply a correction of 0.88 to the a_CDM_(443) data fields prior to assimilation. We recognize this is a potential error, but it is difficult to separate the two in a reflectance inversion methodology because the spectral slopes of absorption are quite similar. The satellite a_CDM_(443) is assimilated with model a_CDOC_(443), which is then easily converted to CDOC using the mass-specific absorption coefficient of CDOC (Yacobi et al., 2003).

Upwelling radiances are not assimilated. They are computed using the distributions of optical constituents in the model, their optical properties (Figure 2), and Equation 16 at 1 nm spectral resolution.

### Model Setup

The model is integrated for 35 years from an initial state using climatological atmospheric forcing, with the new variables PIC and CDOC initialized to 0 concentrations. The model is then run forward in time from 2003 through 2007 using transient atmospheric forcing from MERRA (Rienecker et al., 2011) and assimilating MODIS-Aqua total chlorophyll, PIC, and CDOC.

### Statistical Comparison

The optical constituents of the NOBM-OASIM assimilation model are compared to in situ and/or satellite (MODIS) monthly data where and when available. Phytoplankton groups are compared to in situ data while total chlorophyll, PIC, and a_CDOC_ are compared to satellite estimates. The statistics are aggregated over the 12 basins of the global oceans, mean differences (biases) computed, and then correlations computed over the basins. This provides an estimate of large scale correlations and is very stringent considering the low number of observations. The major ocean basins are divided into 3 main regions, high latitudes (poleward of ± 40° latitude): North Atlantic and Pacific and Southern Ocean, mid-latitudes (between ± 40° and ±10° latitude): North Central Atlantic and Pacific, South Atlantic, Pacific and Indian, and North Indian, and tropical basins (between ± 10° latitude): Equatorial Atlantic, Pacific, and Indian. Comparison of assimilated model results with the data used for assimilation is typically insufficient for assessing assimilation performance (Gregg et al., 2009). However, in this case the objective is to simulate dynamic global water-leaving radiances to support a proposed mission, not to assess the assimilation methodology. Here, knowledge of the biases and uncertainties in the underlying ocean optical constituents derived from the assimilation model is best achieved using the satellite data inputs for assimilation. Normalized water-leaving radiance using OASIM and the computed optical constituent distributions are compared to MODIS at the available MODIS bands, 412, 443, 488, 531, 547, and 667 nm. Using 1 nm upwelling radiances at the center of MODIS bands, we can evaluate the simulated bias and uncertainty with MODIS data and avoid model/data band misalignment. These statistics are not aggregated by basin.

## RESULTS

We evaluate ocean optical constituents, specifically phytoplankton, total chlorophyll, PIC, and a_CDOC_, the latter three of which are provided as data sets from MODIS-Aqua. Water is a constant background and we are not aware of global data on detritus. We evaluate water-leaving radiances by comparing model upwelling radiances at MODIS-Aqua wavelengths with those MODIS-Aqua radiance data.

### Global Ocean Optical Constituents

**FIGURE 4:**
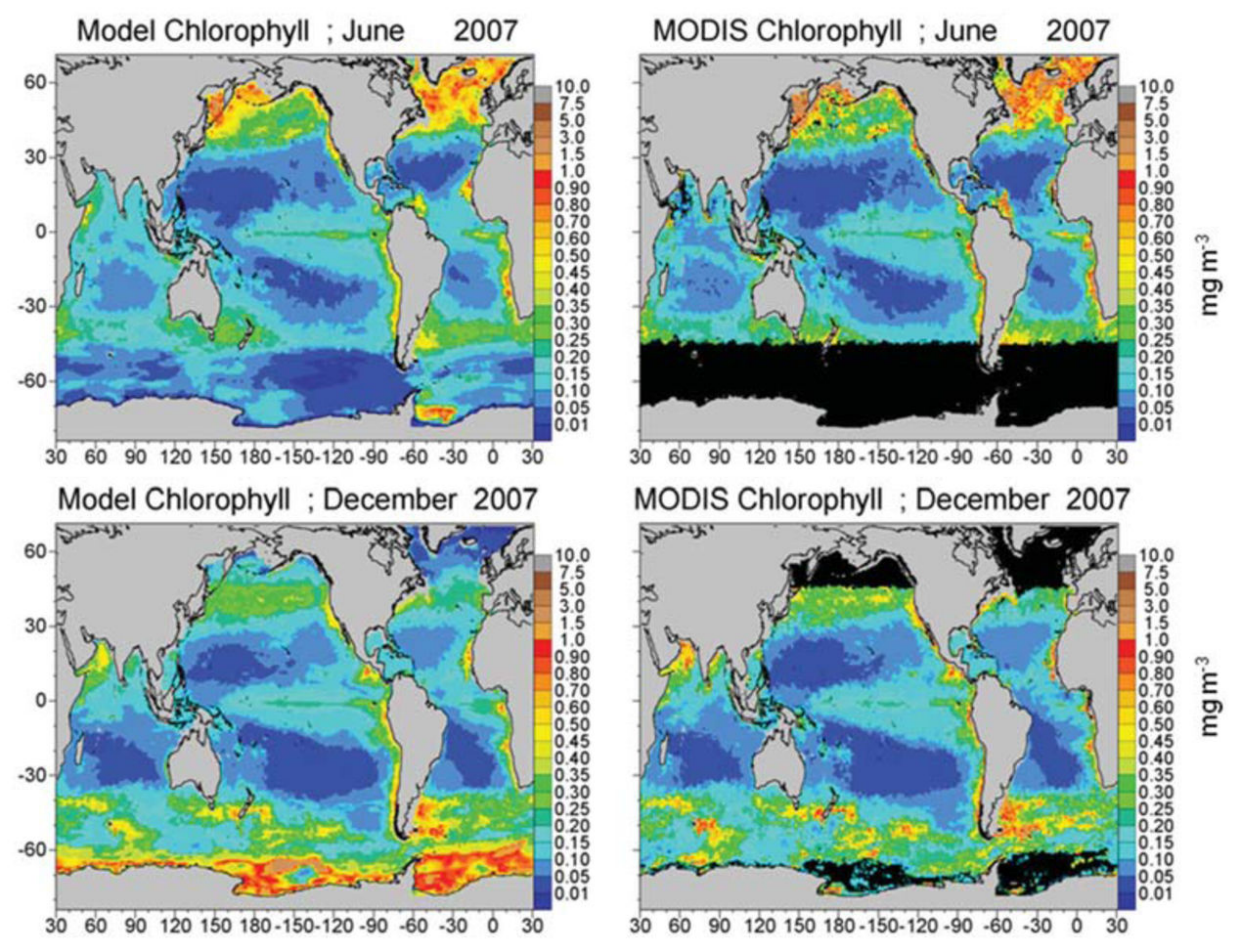
Model assimilated total chlorophyll for June and December 2007 compared to MODIS-Aqua chlorophyll.

Total chlorophyll from the assimilated NOBM-OASIM model is within −35.9% of satellite data (model low), with a correlation across basins of 0.869 (*P* < 0.05; Figure 4; Table 1). The model is low because of uncorrected a_CDM_ in the satellite data, especially near coasts and river mouths, which artificially drives up the estimates of chlorophyll.

Phytoplankton group relative abundances are positively correlated with in situ data for diatoms, cyanobacteria, and coccolithophores (*P* < 0.05) but chlorophytes are not correlated (Table 1). All four groups have relative abundance biases < ±20% compared to in situ data, with diatoms the largest at 17%.

**FIGURE 5:**
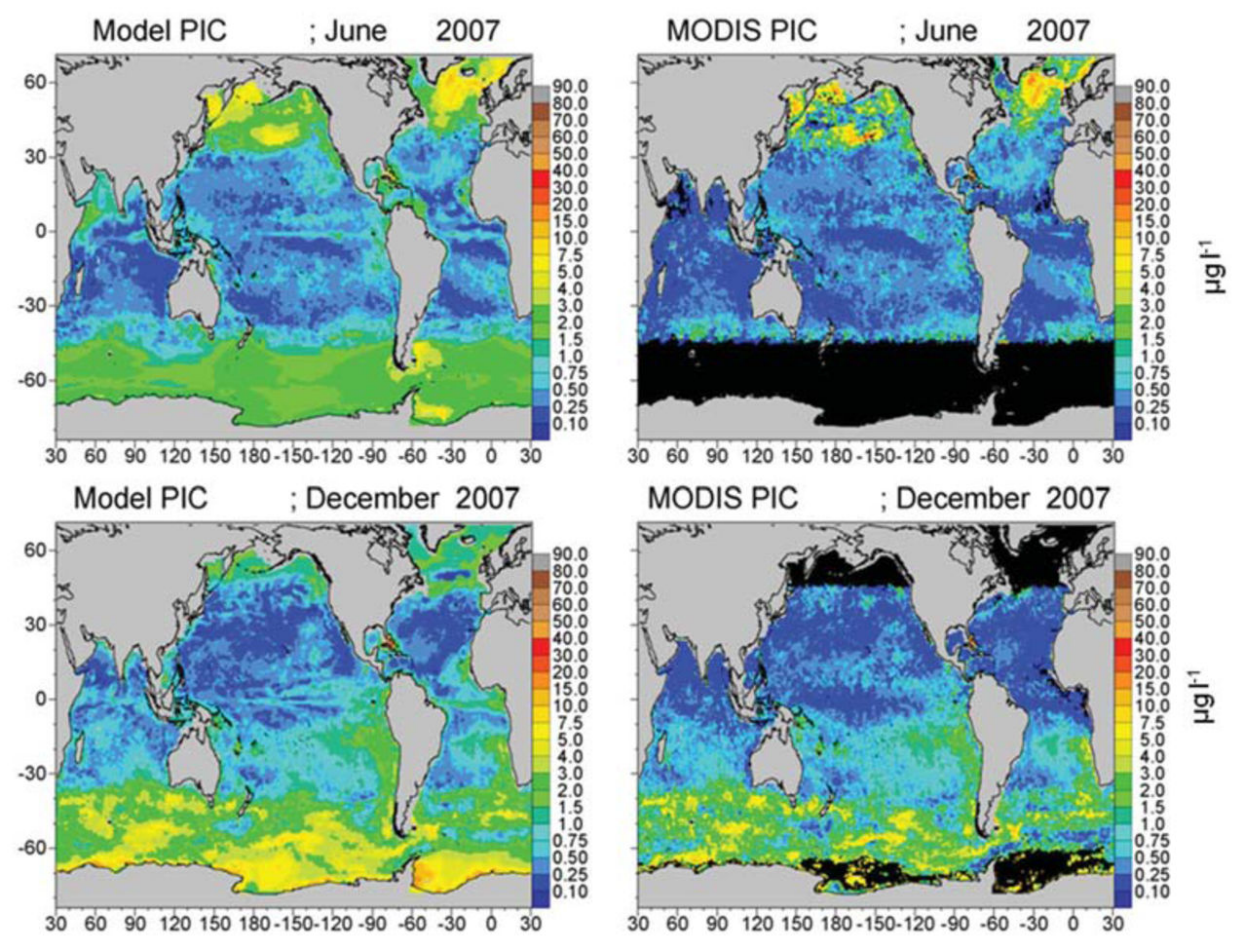
Model assimilated PIC for June and December 2007 compared to MODIS-Aqua PIC.

**FIGURE 6:**
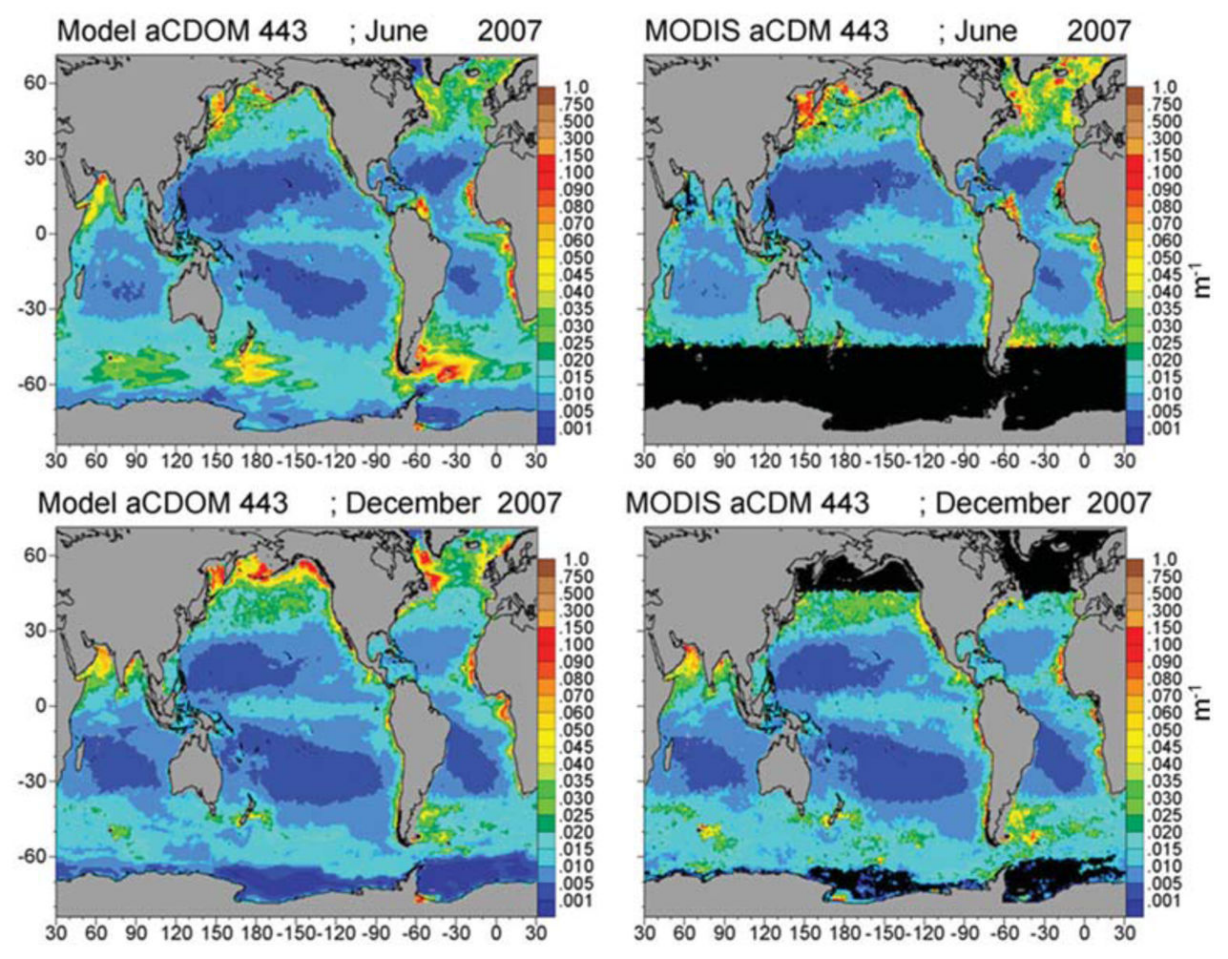
Model assimilated a_CDOC_ 443 nm for June and December 2007 compared to MODIS-Aqua a_CDM_ 443 nm.

Assimilated PIC is correlated with satellite estimates (*P* < 0.05) and concentrations are within −28.5% (Figure 5; Table 1). Simulated PIC is overestimated and more widespread in the Southern Ocean in December, but otherwise exhibits similar variability as indicated by the correlation coefficient (*r* = 0.868). It is unable to capture the localized extreme high concentrations in June in the northern high latitudes, which leads to model underestimates globally. Model comparison of a_CDOC_ (443 nm) is within −24.6% of satellite estimates of a_CDM_ (443 nm) (Table 1), which represents the combined absorption of dissolved matter and particulate matter (detritus). A basin correlation coefficient of 0.890 (*P* < 0.05) is obtained (Table 1). Maps of global distributions for June and December 2007 illustrate the comparison between model and data (Figure 6). Although river discharge is not included in the model, high a_CDOC_ is produced at major river mouths (e.g., Amazon, Orinoco, Congo) via the assimilation of a_CDM_ (see Figure 6).

### Global Normalized Water-Leaving Radiances

**FIGURE 7:**
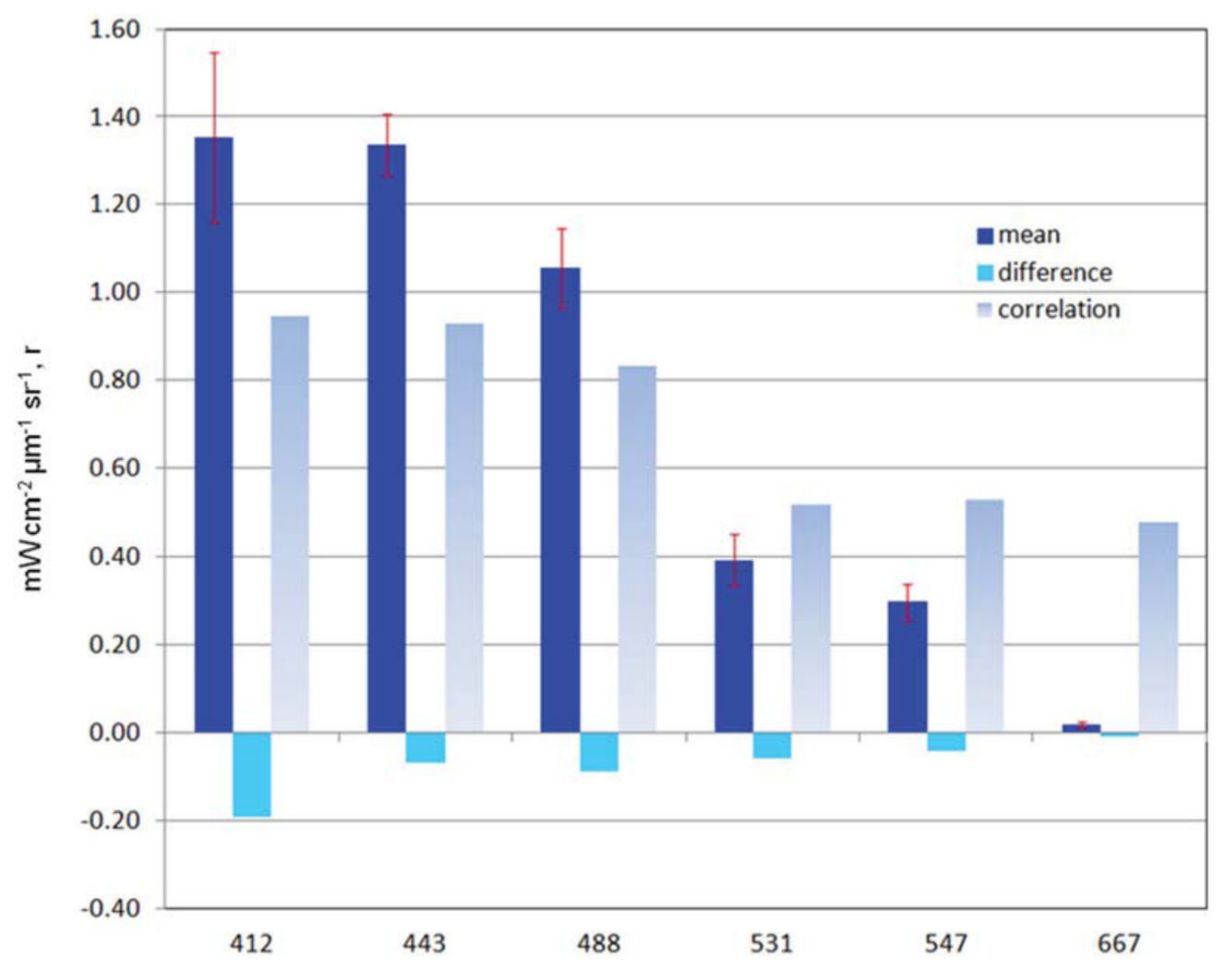
Global statistics on model normalized water-leaving radiances L_w_N(λ) compared to MODIS-Aqua data for 2003–2011 Mean radiance and difference is mW cm^−2^ μm^−1^ sr^−1^. Correlation is *r*-value. All correlations are significant (*P* < 0.05: *N* > 3.7 × 10^6^. Error bars represent semi-interquartile range.

The mean of the global median difference of model normalized water-leaving radiances with MODIS-Aqua radiances for all 6 bands for the period 2003–2007 is −0.074 mWcm^−2^ μm^−1^ sr^−1^ (−10.4%) with a mean semi-interquartile range of 0.077 and a significant correlation of 0.706 (*P* < 0.05). There is a positive and significant correlation with all the simulated radiances with satellite data (Figure 7). The largest relative difference (−30%) and lowest correlation (*r* = 0.48) occurs in the longest MODIS band, 667 nm (Figure 7). Band 1 (412 nm) has the largest absolute difference (−0.19 mWcm^−2^ μm^−1^ sr^−1^; Figure 7), but only the third largest relative difference with a mean of −12.5%, and it has a high correlation of 0.946. All simulated radiances are low relative to data (Figure 7). Correlations of the longer visible wavelengths, 531, 547, and 667 nm are much lower than those of the shorter wavelengths.

**FIGURE 8:**
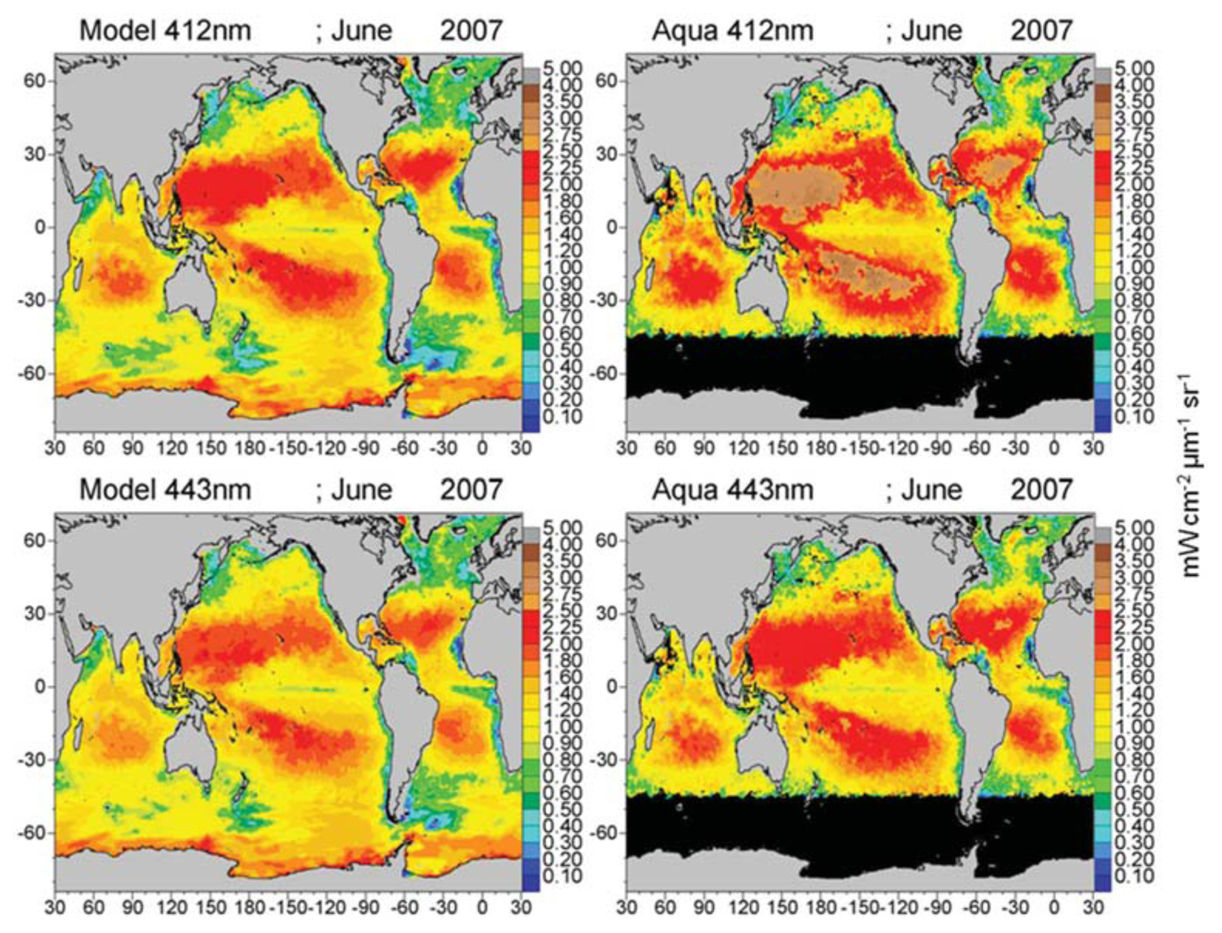
Model normalized water-leaving radiances L_w_N(λ) for 412 and 443 nm compared to MODIS-Aqua radiances.

**FIGURE 9:**
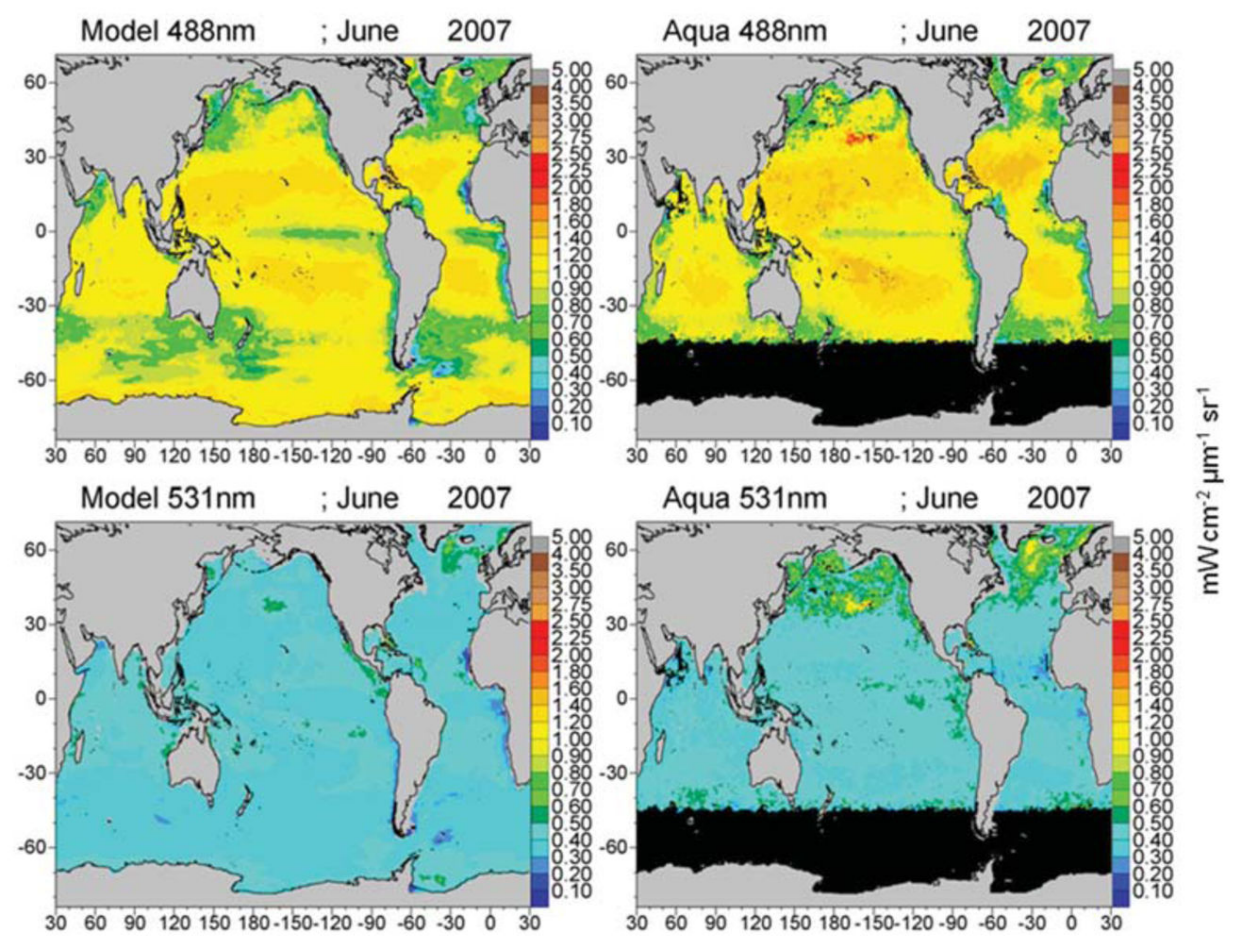
Model normalized water-leaving radiances L_w_N(λ) for 488 and 531 nm compared to MODIS-Aqua radiances.

**FIGURE 10:**
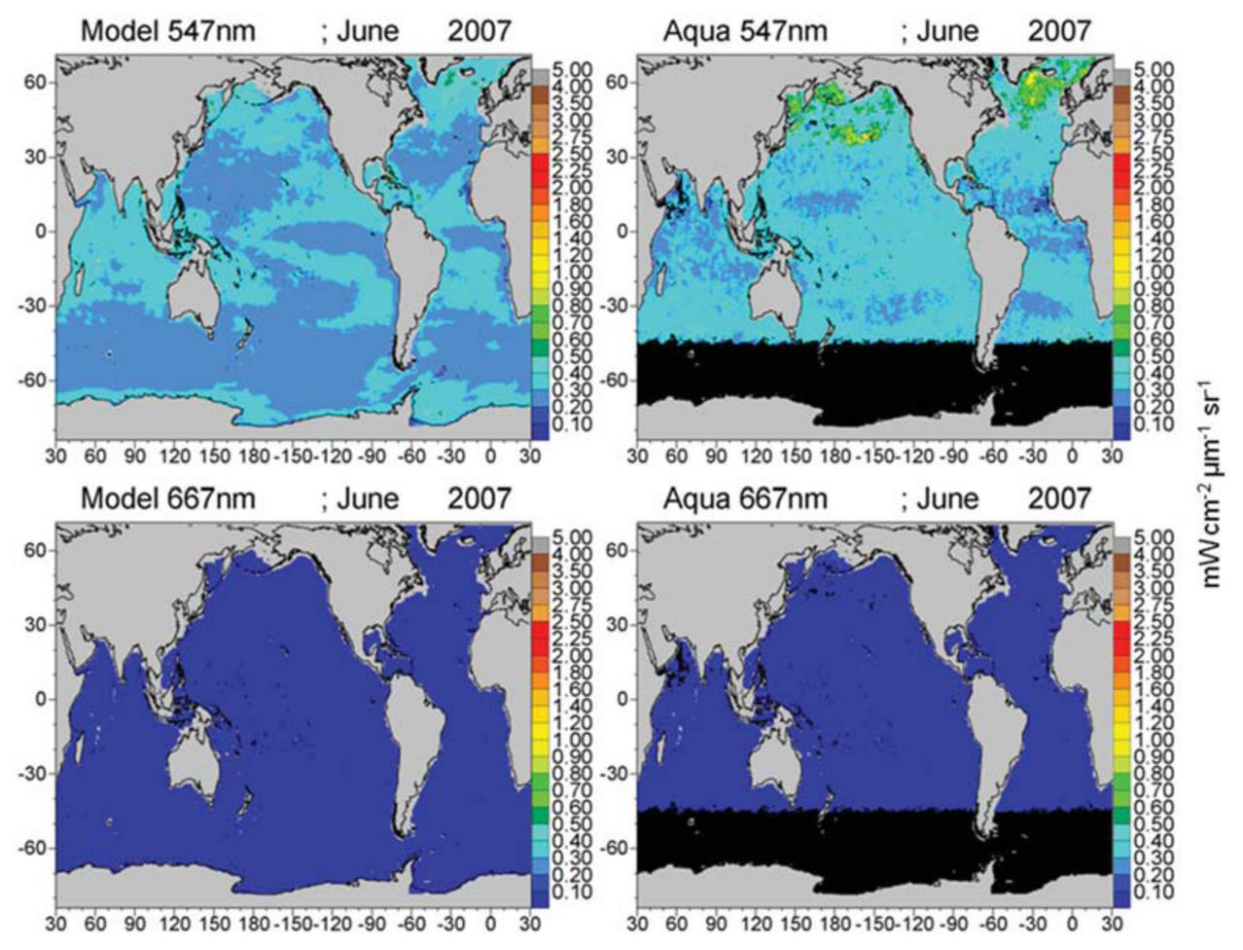
Model normalized water-leaving radiances L_w_N(λ) for 547 and 667 nm compared to MODIS-Aqua radiances.

Global maps of water-leaving radiances illustrate the spatial agreement and discrepancies between the model and satellite data (Figures 8–10). The spatial distributions reflect the biases and correlations shown in Figure 7. Low biases in model radiances are apparent for all bands, but the locations differ. Low model radiances are most apparent for the shorter wavelengths (412 and 443 nm) in the central gyres (Figure 8). Mid-range bands (531 and 547 nm) show low model biases in the northern high latitudes (Figures 9, 10). The longest MODIS band (667 nm) does not exhibit a model bias as shown in Figure 7, but the bias is below the spectral resolution of the figure.

**FIGURE 11:**
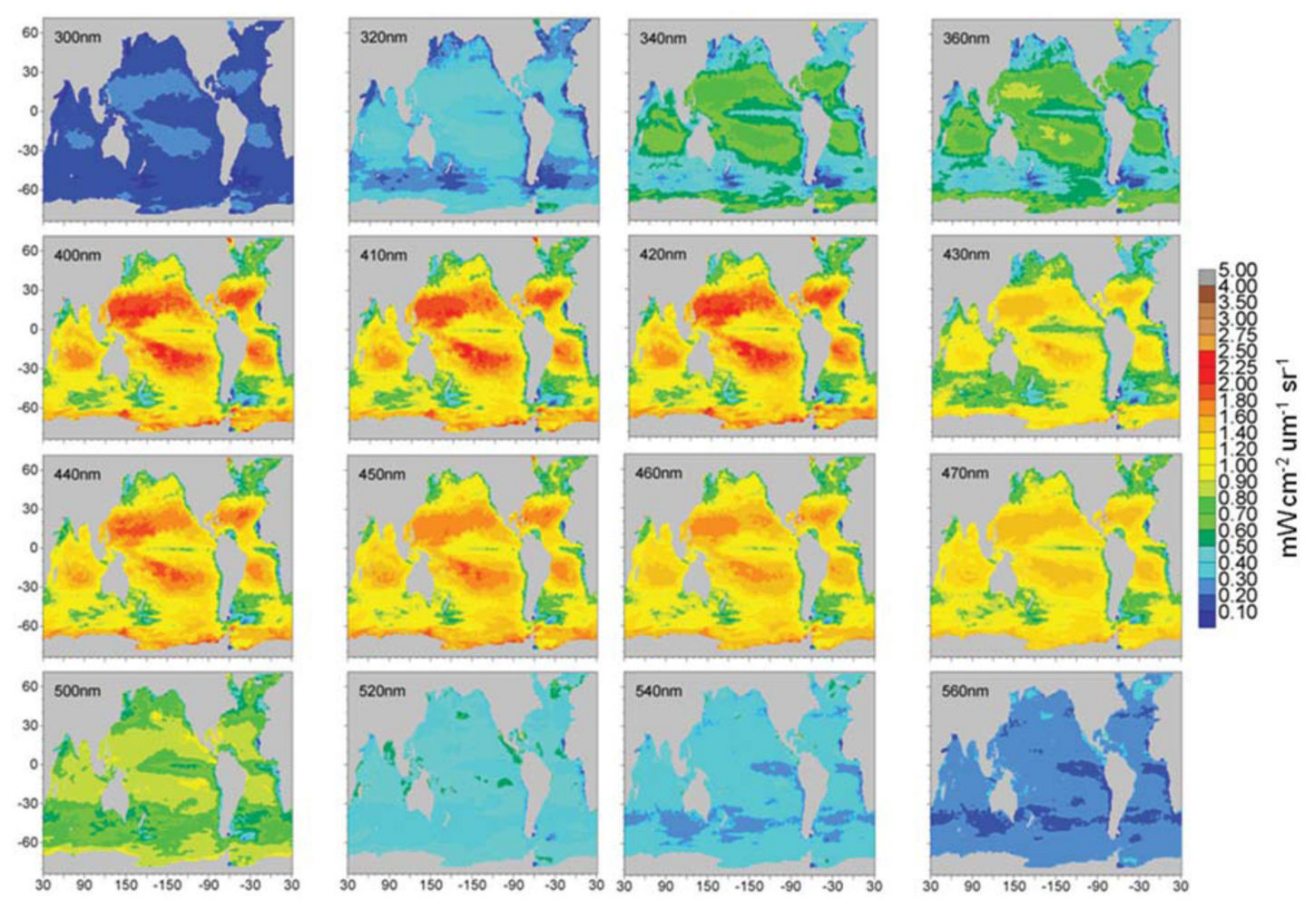
Model normalized water-leaving radiances for selected wavelengths in the ultraviolet and visible region.

**FIGURE 12:**
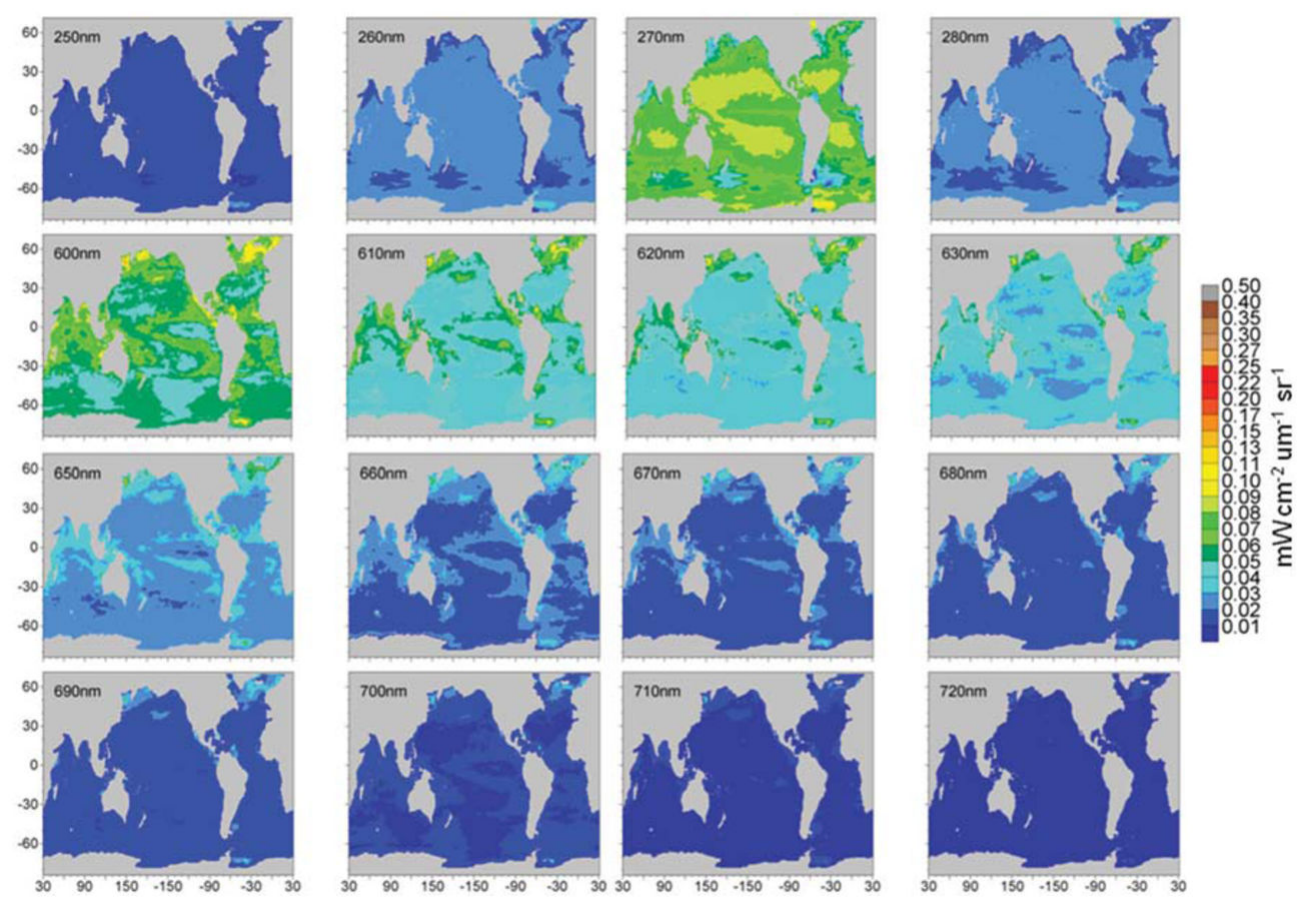
Model normalized water-leaving radiances for selected wavelengths in the ultraviolet, long visible, and near-infrared region Note scale change.

Maps of normalized water-leaving radiances at various wavelengths from the 1 nm hyper-spectral resolution capability are shown in Figures 11, 12. The radiance wavelengths are broken into the two figures to capture variability over the widely-ranging radiance values shown. The second set of radiance maps (Figure 12) uses a different scale for radiance values. Otherwise, spatial variability in these radiances is not visible.

**FIGURE 13:**
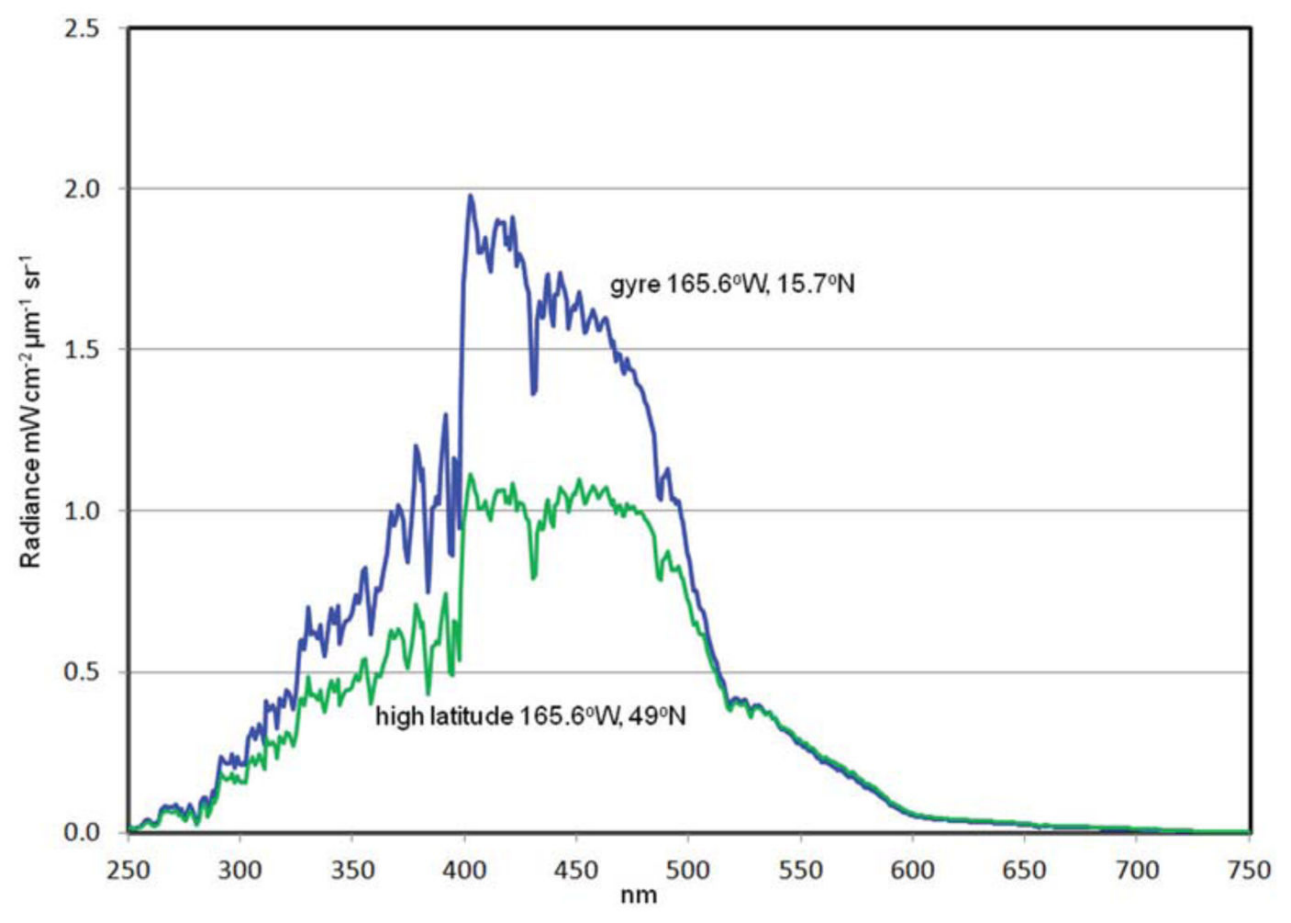
Normalized water-leaving radiances from two locations in the Pacific Ocean: a gyre location (low chlorophyll) and a high latitude location (high chlorophyll).

Two locations in the North Pacific Ocean are selected to show hyperspectral variability in different oceanic environments (Figure 13). One is a low-chlorophyll central gyre location which is characterized by low chlorophyll, PIC and CDOC, southwest of Hawaii. The other is in the high latitude North Pacific just south of the Aleutian Islands, where high chlorophyll, PIC and CDOC prevail. Hyperspectral 1 nm normalized water-leaving radiances show considerable differences in magnitude and local spectral slopes, suggesting the potential for discrimination of ocean constituents from PACE.

## DISCUSSION

We have described a comprehensive model of optical constituents and their influences on hyper-spectral upwelling radiance in the global oceans. The model contains a representation of major optical constituents, namely, water, total chlorophyll, four major phytoplankton taxonomic/functional groups, organic detritus, PIC, and CDOC. All except water are prognostic variables in the model with individual sources and sinks, and with full dynamical capability arising from advection and diffusion processes in the global oceans.

Normalized water-leaving radiances from the global distributions of optical constituents have been quantitatively compared to MODIS-Aqua radiances for the 6 wavelengths available at 412, 443, 488, 531, 547, and 667 nm. These 6 discrete wavelengths provide only a partial basis for estimating the potential of a global dynamical model to represent the hyper-spectral capability of the next generation PACE mission. Thus, the error estimation is incomplete, and relevance to PACE and its ability to simulate future global hyper-spectral radiances is unconfirmed. However, the comparison of the model with the 6 MODIS bands suggests a level of skill sufficient to support some analysis of mission capability and design, and the level of caution necessary to proceed in these activities is quantified here.

### Global Ocean Optical Constituents

The global ocean biology model is optically comprehensive, but it is not complete. There are optical constituents in the oceans that are not included in the model. Some can be important, sometimes globally but most often regionally. For example, bacteria and virus scattering is not present in the model. Bacteria scattering is considered an important component of the scattering from the living part of the particulate pool, possibly dominating the phytoplankton (Balch et al., 2002; Stramski et al., 2004). However, the scattering contributions from the living components are estimated to be small relative to detritus (Stramski et al., 2004). We assume here that bacteria covary with detritus. Virus scattering is disputed. Balch et al. (2002) suggest it may be important while Stramski et al. (2004) consider it negligible.

Minerals/suspended sediments are not included. These are most important near river mouths at times of high discharge, but they also occur from particulate deposition from the atmosphere, such as desert dust (Wozniak Stramski, 2004) or organic carbon from biomass burning. Absorption by mycosporine-like amino acids (Moisan and Mitchell, 2001) is not included in the model. This is most important in the ultraviolet spectrum, and casts suspicion on the simulated representations of water-leaving radiances in this spectral region by the model. PACE is nominally expected to detect as low as 350 nm (PACE Mission Science Definition Team Report, 2012), but there may be interest in expanding that range if it is technically and economically feasible. The most recent configuration concept is to expand the detection limit to 320 nm. Inclusion of the effects of mycosporine-like amino acids should be included in future improvements of the biological global model.

Finally, four phytoplankton groups cannot possibly represent the range and complexity of the phytoplankton taxa living in the oceans. Unfortunately, detailed knowledge of the optical, physical, and physiological properties of the world’s ocean phytoplankton, which is required to parameterize our coupled optical, physical, and biological model, is not available. We recognize our four groups as a shortcoming, but they do capture a substantial range of functionality. Diatoms represent the fast growing, fast sinking component particularly important in the carbon and silicon cycles. Cyanobacteria represent the functional opposite, as a slow growing, nearly floating, very small phytoplankton that occupy the nutrient-desolate vast ocean gyres, and additionally have a limited nitrogen-fixing capability (Rousseaux et al., 2013). Coccolithophores represent a unique category of calcium-producing phytoplankton, which scatter light out of the oceans effectively and play a role in the carbon cycle by affecting alkalinity in addition to photosynthesis and respiration processes. Finally, chlorophytes represent (or at least are intended to represent) intermediate phytoplankton with characteristics between diatoms and cyanobacteria. It is this intermediate category that is most under-represented here and is where much of the diversity of the global ocean arises.

The fact that chlorophytes are not significantly correlated with in situ data in the model is particularly important because they are the only group in the model representative of the diverse phytoplankton component between the functional extremes of diatoms and cyanobacteria, save for the unique coccolithophore class. This is a deficiency in the model as it pertains to PACE and we acknowledge that their lack of correlation with data is important. However, in the model we assume chlorophytes represent a very wide range of phytoplankton, often reported to as nanoplankton. Since in situ data sets rarely specifically identify chlorophytes, we compare our model chlorophytes to in situ data reports of nanoplankton, non-diatoms or non-pico-prokarytotes, representing this middle ground between diatoms and cyanobacteria. We note that most of the lack of correlation with in situ data occurs in the high latitudes, where chlorophytes are not common, but other types on nanoplankton are sometimes abundant. The abundance of these reported nanoplankton in the high latitudes, coupled with the near-absence of chlorophytes in the model, is the cause of the lack of correlation. The model representation of chlorophyte abundance corresponds much more closely with reported observations of nanoplankton in the lower latitudes, suggesting that simulation of PACE radiances in these basins is likely to be more realistic.

### Using Data Assimilation to Improve the Representation of Global Optical Constituents

The assimilation of chlorophyll has been demonstrated to improve the representation of distributions regionally and globally (Hu et al., 2012; Fontana et al., 2013; Gregg and Rousseaux, 2014). Assimilation of PIC and a_CDM_ has not been attempted globally, to our knowledge. Our purpose in assimilating PIC and a_CDM_ is not novelty but fidelity. The optical properties of PIC have been established (Balch et al., 1996; Gordon et al., 2009) and one can find models of production and dissolution in the literature (Buitenhuis et al., 2001; Gangsto et al., 2011; Barrett et al., 2014). Our parameterization of sinking processes is a matter of trial and error using global satellite fields of PIC from MODIS-Aqua. Assimilation of a_CDM_ is a larger challenge. Although assimilation of optical properties, in particular the diffuse attenuation coefficient, has shown value (Ciavatta et al., 2014), the assimilation of a_CDM_ is more problematic because there a few examples of its use in coupled physical-biogeochemical models (e.g., Buitenhuis et al., 2001; Xiu and Chai, 2014; Dutkiewicz et al., 2015) We approach the problem in a bottom-up fashion, adding a dynamical tracer to the biogeochemical model suite, i.e., CDOC, which has the optical properties of a_CDOC_(λ). The characterization of the biological production and loss terms for CDOC is more or less straightforward, as it can be related to those from the optically inert DOC (e.g., Aumont et al., 2002). Loss of CDOC via the absorption of spectral irradiance is more difficult. Although the absorption characteristics are well-established, how that relates to CDOC concentration and subsequent destruction is difficult to quantify. There is regional information on defining a quantum yield for CDOC photolysis, φ_cdoc_ (e.g., Reader and Miller, 2012, 2014), but we require a global spectrally integrated solution. We consider our parameterization of φ_cdoc_ to be tenuous, but we take consolation that the assimilation guides us to a reasonable result in the end, and even rectifies the absence of river input in the model, which is a major source of CDOC to the oceans. For the present purpose of providing a model to assist in the early stages of development of a future mission, we believe our approach has support as an initial step. The statistical comparison of CDOC distributions with satellite data supports this approach as well (Table 1; Figure 6).

### Global Water-Leaving Radiances

The comparison of model water-leaving radiances with MODIS-Aqua at the 6MODIS bands suggests some skill in the simulation: the mean of the global median difference is −0.077 ± 0.079 mW cm^−2^ μm^−1^ sr^−1^ (−10.4%). A statistically significant correlation with all the simulated radiances with satellite data is found (Figure 7), although some of the correlation coefficients are low. We emphasize that the radiances are not assimilated. We emphasize that the radiances are not assimilated. Rather, they are the result of the distribution of optical constituents in the coupled model.

The longer visible wavelengths, 531, 547, and 667 nm have lower correlations with satellite data than the shorter ones. There is much less spatial variability in the longer wavelengths (Figures 9, 10). Ocean color sensors have much larger uncertainty in these wavelengths (Mélin et al., 2016) which contributes to the decrease in correlation of these radiances here.

The model is always low relative to the MODIS normalized water-leaving radiances. The low model radiances occur in different regions for the different bands. For the shortest MODIS wavelengths, 412 and 443 nm, largest biases occur in the ocean gyres (Figure 8), where ocean biological optical constituents are at their lowest magnitudes. The 412 nm band has a larger model-data discrepancy than the 443 nm band (Figure 7). For the midrange bands 531 and 547 nm, the model-data discrepancies occur in the northern high latitudes.

The model low bias for L_w_N(412) and L_w_N(443) in the central gyres suggests either missing scattering in the model or overestimated absorption. These regions are biologically the most barren regions in the global ocean, where the main optical constituent is water. The southeast Pacific gyre has been the subject of an intensive field campaign (BIOSOPE), and several investigators have relied upon this data set to revise the understanding of the optical properties of seawater (Morel et al., 2007; Lee et al., 2015), CDOM and particulate detrital absorption (Bricaud et al., 2010), and total particulate backscattering (Twardowski et al., 2007). The Lee et al. (2015) seawater absorption revision reduced the absorption coefficients, thus producing more scattering, which has helped in our model here, since the revision is used in our calculations. Residual underestimation of scattering and/or overestimation of absorption still prevails in the simulation.

It is possible that the exclusion of mineral scattering in the model is important in the central gyres. However, this argument would be more persuasive for the North Central Pacific and North Central Atlantic gyres than the South Pacific gyre, since there are few atmospheric depositions to this region. One cannot neglect the possibility of radiative model error as well. Perhaps the use of empirical constants in a remote sensing reflectance algorithm, such as Lee et al. (2002) or Gordon et al. (1988), would improve radiances. However, this would sever connections in the radiative modeling system, which uses an analytical model for simulation of both irradiance transmittance in the ocean and the irradiance and radiance re-emerging to and above the surface.

Finally, the spectral slope of detrital absorption S_d_(λ) used here, 0.013 nm^−1^, which was derived from assessment of small particulates in the Chesapeake Bay (Gallegos et al., 2011), is higher than that derived from the southeast Pacific by Bricaud et al. (2010), 0.0094 nm^−1^. This could lead to the higher absorption and subsequent lower backscatter, especially in the shorter wavelengths, as we observe here. How much will depend upon the concentration of detritus in this region and the other central gyres.

The model also exhibits low radiances compared to MODIS for the 531 and 547 nm bands (Figures 9, 10), except these are mostly located in the northern high latitudes. These discrepancies appear to be related to the distributions of PIC (Figure 5). Model PIC distributions here largely correspond with satellite distributions, although local maxima in the southern central North Pacific and the Greenland Sea are subdued in the model (Figure 5). These two locations are responsible for the largest disagreements. However, additional local maxima in satellite PIC occur in the northern Bering Sea and western Sea of Okhotsk (Figure 5), that are not accompanied by high water-leaving radiances in the MODIS 531 and 547 nm bands (Figures 10, 11). High chlorophyll (Figure 4) and a_CDOM_ (Figure 6) in the model and MODIS likely suppress the scattering of PIC in the northern Bering Sea and Sea of Okhotsk. But the lack of representation of the high scattering by PIC in the south-central North Pacific and Greenland Sea results from the spatially smoother PIC distributions in the model compared to MODIS (Figure 5). Overall widespread higher radiance dispersed throughout the northern basins in likely due to inadequate PIC scattering in the model, considering the correspondence between model and satellite PIC distributions. Excessive absorption by other constituents in the model can contribute to the differences in radiances between model and data here. Such high absorption would likely be due to phytoplankton (particularly diatoms, which are predominant in the North Pacific), or coccolithophores which are prevalent in the North Atlantic.

Global maps of selected normalized water-leaving radiances other than those coincident with MODIS-Aqua show considerable spectral and spatial variability (Figures 11, 12). The figures are divided into two groups because the spectral range is so large that different scales must be utilized. Figure 10 shows radiances from two ultraviolet-b bands (300 and 320 nm), to an ultraviolet-a band (340 nm), and 13 through mid-range visible (360–560 nm). There is a steady increase in radiance intensity as we progress from shorter to longer wavelengths until about 400–410 nm, then a slow decline to 560 nm. An exception to this trend is the radiance at 430 nm, which shows a sharp decline relative to its neighbors at 420 and 440 nm (Figure 11). This is due to a local minimum in the extraterrestrial irradiance that is employed at 1 nm resolution (Thuillier et al., 2004). These local minima and maxima occur occasionally in the radiance spectrum and represent a potential issue when choosing band locations for PACE. There can be very large swings in signal strength in short wavelength segments.

The second selection of radiance wavelengths, at extreme ultraviolet-b along with the long end of visible and 3 near infrared wavelengths (Figure 12), shows increasing intensity from 250 through 270 nm, and another from 600 to 630 nm, before reversing from 650 to 720 nm. There is very little normalized water-leaving irradiance at 720 nm and spatial variability will require another scale change to be visible. There is another anomaly, this time a local maximum, at 270 nm, again due to the high spectral variability in the extraterrestrial irradiance. This set of radiances, with the possible exceptions of the shorter 600 nm bands, suggests that ocean signal detection from a satellite will be challenging. The longer 600 nm wavelengths are conventionally used for atmospheric correction since there is so little ocean contribution to the normalized water-leaving radiance (e.g., Gordon, 1997) while NIR bands (e.g., Wang et al., 2016) have shown additional promise for the rare conditions when the ocean does contribute here.

### Potential Uses for Pace Mission Design and Analysis

The hyper-spectral 1 nm resolution ocean model presented here suggests skill for simulating global normalized water-leaving radiances, as shown by the comparison with the moderate resolution bands for MODIS-Aqua. Quantitative error characterization shows the limits of usefulness in the MODIS bands and the potential for simulating radiances outside the current satellite observational capability. This suggests at least some usefulness for pre-launch PACE design and analysis activities, guided by due caution of the limits of the simulation.

Representation of remotely-sensed normalized water-leaving radiances may be approached using airborne (e.g., Airborne Visible/Infrared Imaging Spectrometer, Portable Remote Imaging SpectroMeter), or in situ data, or coastal spaceborne imagers, such as the Hyperspectral Imager for the Coastal Ocean. However, the global observing simulation capability of the present assimilated model can contribute in other important ways that airborne, in situ and limited spaceborne data cannot.

The most important attribute that separates PACE from previous ocean color missions is its global hyper-spectral resolution capability. The global simulation described here at 1 nm can help clarify questions about band selection, specifically choice of bands, band widths, number of bands and their center location. Variability over orbital tracks encountering a range of solar and satellite angles complicates band selection decisions in ways that in situ and most airborne activities cannot resolve. The global seasonal nature of the simulation assists in understanding potential signal strength issues over the diverse regions and seasons encountered in a global mission. It is possible to sample the simulated 1 nm bands in various scenarios to observe and optimize their location and widths, subject to the viewing constraints of an orbiting platform. Optical effects, such as spectral response function can be included in the analysis. As mission design and construction proceeds, issues can arise and tradeoffs must be assessed. These often include signal-to-noise ratios, detector saturation effects, gain selection and operation (if applicable), stray light, and bright target recovery. The existence of the simulation described here can provide numerical answers from an orbital perspective, even if approximate, as these issues emerge. The limitations of the model are quantitatively characterized here and can be factored into the decisions on how to proceed. A much more modest simulation, using only a single global map of ocean color data derived from the entire CZCS mission (Gregg et al., 1997), proved helpful in designing and managing the SeaWiFS mission, which, like PACE, had no global observational precedent.

The second most important feature of this simulation is to provide a platform for algorithm development activities. Although the phytoplankton differentiation in the model is necessarily simplified, it can be used in coarse algorithm activities. At worst, algorithms that cannot differentiate among the simple phytoplankton assemblage in the simulation would likely have difficulties in actual ocean observations, where the phytoplankton diversity is enormous.

The simulation can also assist in studies of data collection strategies on orbit. Seasonal variability in phytoplankton/PIC/CDOC distributions is explicitly incorporated in the simulation to include a full representation of optical combinations as seen to date with current missions. If coupled with a similarly comprehensive and hyper-spectral atmospheric simulation, and an orbital viewing platform, the combined models can be used to explore signal retrieval at the sensor and help maximize the ability to meet the challenging goals of this ambitious mission.

## Figures and Tables

**TABLE 1:** Comparison of simulated optical constituents in NOBM-OASIM with data (*in situ* or satellite).

Optical constituent	Difference	Correlation	N (ocean basins)
Diatoms	17.0% (*in situ*)	0.890 *P* < 0.05	11
Chlorophytes	−16.2% (*in situ*)	−0.318 NS	10
Cyanobacteria	−2.4% (*in situ*)	0.732 *P* < 0.05	11
Coccolithophores	5.3% (*in situ*)	0.716 *P* < 0.05	10
Total Chlorophyll	−35.9% (satellite)	0.869 *P* < 0.05	12
PIC	−28.5% (satellite)	0.868 *P* < 0.05	12
a_CDOC_	−24.6% (satellite)	0.890 *P* < 0.05	12
Detritus	NA	NA	NA

NS indicates not significant at 95% confidence. NA indicates data not available for comparison. The satellite comparison uses MODIS-Aqua and model data used are co-located and coincident with monthly mean MODIS data.

## Abbreviations

a_CDM_: absorption coefficient of Chromophoric Dissolved and particulate organic Matter
a_CDOC_: absorption coefficient of CDOC
BIOSOPE: Biogeochemistry and Optics South Pacific Experiment
CDOC: Chromophoric Dissolved Organic Carbon
CZCS: Coastal Zone Color Scanner
DOC: Dissolved Organic Carbon
EnMAP: Environmental MAPping and Analysis Program
GMAO: Global Modeling and Assimilation Office
MAP: Modeling, Analysis and Prediction
MERRA: Modern-Era Retrospective Analysis for Research and Applications
MODIS: MOderate Resolution Imaging Spectroradiometer
NIR: Near InfraRed
NOBM: NASA Ocean Biogeochemical Model
OASIM: Ocean-Atmosphere Spectral Irradiance Model
PACE: Plankton, Aerosol, Cloud and ocean Ecosystems
PIC: Particulate Inorganic Carbon
PRISMA: PRecursore IperSpettrale della Missione Applicativa
PSU: Practical Salinity Units
SeaWiFS: Sea-viewing Wide Field-of-view Sensor
S-NPP: Suomi National Polar-orbiting Partnership
